# Expanding the ABCC-tool for osteoarthritis: Development and content validation

**DOI:** 10.1016/j.ocarto.2024.100488

**Published:** 2024-05-15

**Authors:** V.H.J. Debie, T.A.E.J. Boymans, R.P.G. Ottenheijm, O.C.P. van Schayck, A.H.M. Gidding-Slok

**Affiliations:** a Department of Family Medicine, Care and Public Health Research Institute (CAPHRI), Maastricht University, Maastricht, the Netherlands; b Department of Orthopedic Surgery, Care and Public Health Research Institute (CAPHRI), Maastricht University Medical Center, Maastricht, the Netherlands

**Keywords:** Osteoarthritis, ABCC-tool, Burden of disease, Patient-centered care, Patient reported outcome measures, Shared-decision making

## Abstract

**Objective:**

Osteoarthritis (OA) care should be more person-centered based on physical, emotional and social aspects, instead of the current stepped-care approach solely based on physical symptoms, according to OA patients. By developing a novel module for OA in the Assessment of Burden of Chronic Condition (ABCC)-tool, a tool based on these three aspects, experienced quality of OA care and shared-decision making are expected to improve.

**Design:**

The development of the novel OA module involved a triangular iterative process, interviewing OA patients and healthcare professionals in the field of OA, an expert panel and a literature search to identify the needs to improve OA care. Patients provided feedback on the first version of the OA module, leading to a second version. This second version was used to evaluate content validity. OA patients and healthcare professionals in the field of OA were asked to evaluate relevance, comprehensiveness and comprehensibility, based on the COSMIN methodology. For healthcare professionals, the item-content validity index (I-CVI) was calculated.

**Results:**

The module includes questions about pain, kinesiophobia and joint stiffness. For all three questions, 94% of the patients found these questions important for patients with OA. The I-CVI scores of the healthcare professionals ranged from 1.0 (pain, kinesiophobia) to 0.75 (joint stiffness).

**Conclusion:**

A novel, condition-specific OA module is developed for the ABCC-tool, as a supplement to the generic questions. The module includes three questions, to measure OA specific complaints. This novel module is intended to make the ABCC-tool more elaborate and useable for a larger population.

## 1 Introduction

Osteoarthritis (OA) is a leading cause of disability worldwide, with a burden projected to increase over the coming decades [1,2]. The prevalence is expected to expand due to the aging population, as advanced age is a significant risk factor [[1], [2], [3]]. The disease progresses slowly, but the majority of affected patients experience chronic pain, which can lead to disability, and a reduction of quality of life (QoL) and participation in social activities [[4], [5], [6], [7], [8], [9]]. The symptoms and the severity of symptoms vary among patients, affected joints, and over time due to fluctuations in burden of OA [1,10]. The burden of OA may even worsen when the patient has comorbidities, e.g. diabetes mellitus type II (DM2), which is not uncommon in OA patients [11]. Patients with OA and comorbidities have more limitations in daily life activities, a lower QoL, more pain and a higher risk for depression than OA patients without comorbidities [12,13].

Standard OA care consists of a stepped care approach to improve physical burden, consisting of non-pharmacologic care (e.g. encouraging a healthy lifestyle), pain medication (e.g. paracetamol, NSAIDs, or corticosteroid injection), and surgical treatment (e.g. arthroplasty). Depending on the step in this process, patients with OA are managed by their general practitioner (GP), physical therapist or orthopedic surgeon [5,14]. However, both OA patients and healthcare professionals (HP)s have expressed their dissatisfaction about OA care as it is not personalized and often insufficient. GPs mainly focus on lifestyle changes and physical burden, while patients are unwilling to change their lifestyle due to poor understanding of their condition. Patients prefer to receive sufficient information on pain management, fear of disability, and disease progression to increase self-management and coping [7,[15], [16], [17], [18], [19], [20]]. Moreover, lifestyle changes are also the cornerstone for managing most comorbidities, however, the symptoms of OA often make lifestyle changes hard to achieve. This dissatisfaction, together with the high prevalence of OA and comorbidities, and differences in burden between OA patients, it seems necessary to create a more personalized approach based on shared decision-making to provide patients with the most optimal care [1,10,11,21,22].

The Assessment of Burden of Chronic Conditions (ABCC)-tool can be used as a valuable resource to make shared decision-making possible [21]. The ABCC-tool is developed in line with the definition of health, according to the World Health Organization; “health is a state of complete physical, mental and social well-being and not merely the absence of disease or infirmity” [23]. The tool, developed in 2020 for asthma, chronic obstructive pulmonary disease (COPD), DM2, and chronic heart failure, measures the patients' total health status based on their input, and assesses various aspects, including symptoms, emotions, physical limitations, social experiences, and burden of medication use [21,24].

**Fig. 1:**
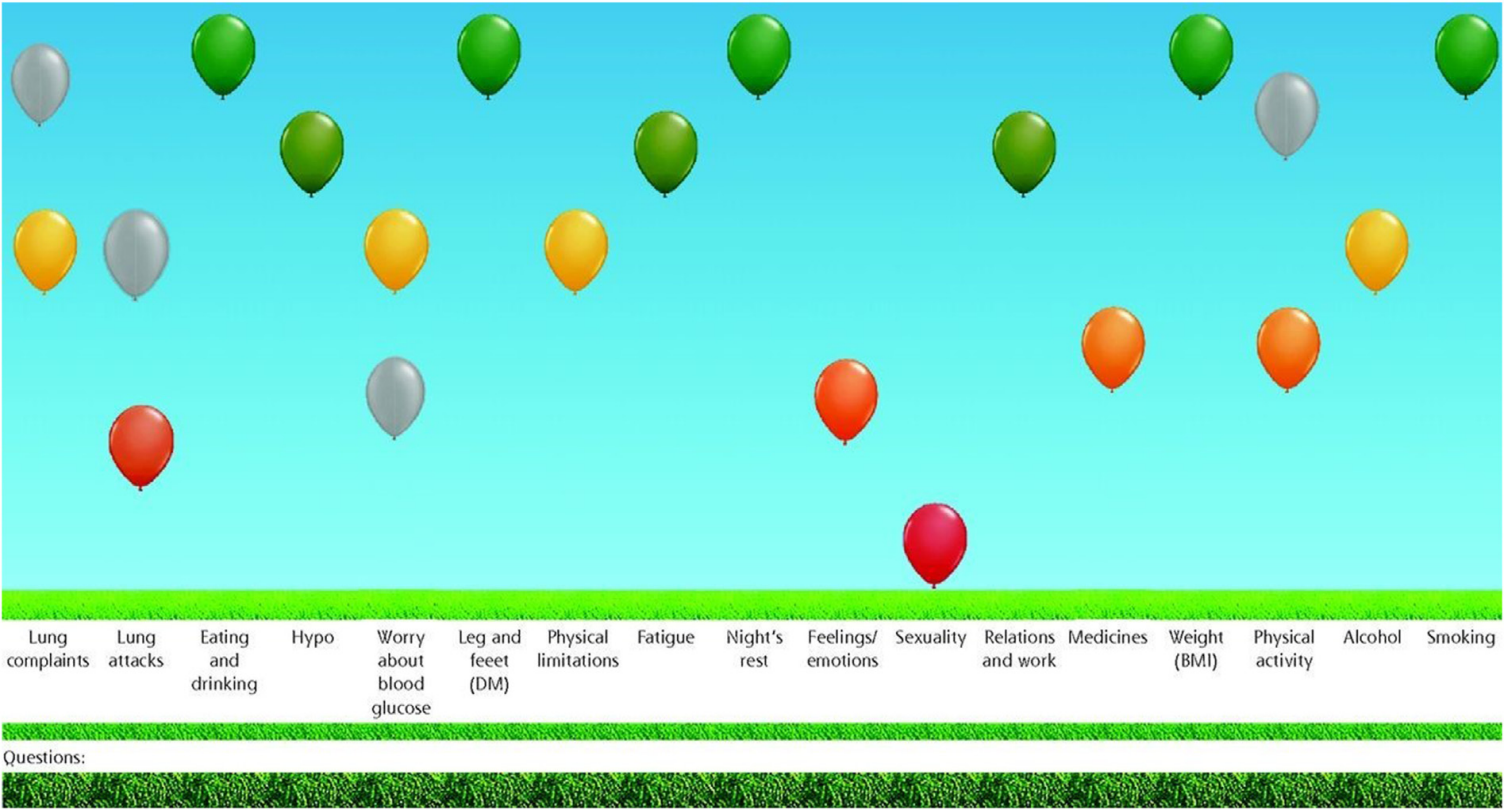
Visual representation of the health status of a patient with COPD and DM2 with the ABCC-tool [24]. The first six balloons represent disease specific domains. The other eleven balloons are based on the questions of the generic and lifestyle module.

The ABCC-tool has a generic and lifestyle component for all chronic conditions, supplemented by condition-specific modules, which can be combined in case of comorbidity [21]. The ABCC-tool portrays a visual representation of each item with balloons, as displayed in Fig. 1. Green and high balloons indicate a higher health status, while red and low balloons indicate a lower health status. Balloons colored yellow or orange are scores falling in between. Grey balloons indicate previous results to show evolutions over time [25]. To create a personalized treatment plan, these balloons support a shared decision-making conversation between the patient and HP by showing a pop-up with treatment advices (based on GP and medical specialist guidelines) relative to the burden when clicking on a specific balloon [25].

The ABCC-tool aligns with the vision that OA care should be person-centered and based on physical, mental, and social well-being [21]. Furthermore, the ABCC-tool facilitates making a personalized treatment plan, primarily driven by the patient's input, and based on the treatment recommendations [21]. Person-centered care increases physical and social well-being, enhances patients' self-management skills, improves health outcomes and increases patients' satisfaction about healthcare [26,27]. This approach enhances patient motivation and self-management, and supports patients with OA to have greater control over their chronic condition and QoL [21]. A previous effectiveness study with the ABCC-tool in patients with COPD, asthma, DM2 and chronic heart failure shows an improvement in patient activation and experienced quality of care [28]. With the previous beneficial effects of the ABCC-tool on managing chronic conditions [21,25,28], we assume that extending the ABCC-tool for OA patients might have comparable effects. Therefore, it is desired to develop a condition specific ABCC-tool module for OA. This study describes the initial phases including the development and content validation of the novel OA module of the ABCC-tool.

## 2 Methods

### 2.1 Development

The novel OA module for the ABCC-tool is developed in a triangular iterative process, to be used as complementary condition-specific module next to the generic and lifestyle questions of the ABCC-tool. The development consisted of individual interviews with patients with OA and HPs in the field of OA, an expert panel, and a literature search. Patients with OA evaluated the initial draft, with back-and-forth interactions throughout this process.

HPs in the field of OA (i.e. physical therapists, GPs, orthopedic surgeons, rheumatologists, and rehabilitation physicians) and patients with OA were invited for individual interviews between April and July 2021, both from a university medical center (UMC). Inclusion criterion for patients was to have OA in at least one joint, regardless of which joint was affected. There were no exclusion criteria. The interviews were based on a topic list, which was adjusted in between interviews to ensure all topics were covered. The aim of the interviews with patients was to explore the burden of disease to develop a first version of the OA module of the ABCC-tool from a patient's perspective. The aim of the interviews with the HPs was to gain more insights into the needs and expectations of OA patients from HPs. We aimed to interview ten to fifteen patients with OA and ten to fifteen HPs in the field of OA, or until data saturation was reached. All interviews were audiotaped, transcribed verbatim and analyzed in NVivo (Version 12. Lumivero. Denver, Colorado, USA).

Next, two meetings with an expert panel, including one specialized GP (also known as GP with extended role in MSK disorders), one rheumatologist, one orthopedic surgeon, one GP, one physical therapist, one chronic pain specialist, six researchers, and one patient, were organized to discuss the results of the interviews, and to give an advise on what topics should be included in the OA module of the ABCC-tool. The panel discussed five topics: self-reliance, pain, joint stiffness, kinesiophobia, and work. The point of view of all experts was summarized per topic. Thereafter, a conclusion per topic was drawn of which all members of the expert panel agreed on.

Besides, a literature search was performed on PubMed and Google Scholar to find questionnaires that aim to measure burden of hip, knee, foot/ankle, and hand/wrist OA. In addition, trustworthy Dutch websites that provide information about OA, such as the website of the Dutch Arthritis Foundation (ReumaNederland) and the Dutch College of General Practitioners (DCGP (in Dutch: NHG)), were also used to gather information about the burden of OA. ReumaNederland is a Dutch health fund and patient advocacy group dedicated to patients with various rheumatic diseases, including OA. The DCGP issues guidelines for various musculoskeletal disorders, including OA [[29], [30], [31], [32]]. Questionnaires and websites were scanned to see if important aspects related to the experienced burden were missed.

Combining the results of the interviews, expert panel and literature search, we drafted a first version, which we presented to at least ten patients with OA at an orthopedic outpatient clinic to receive feedback on the questions (comprehensiveness, comprehensibility) and answer options. In case of linguistic ambiguities or the absence of important questions, the draft was reconsidered and adjusted to the patients’ needs. Data will be presented in an integrated manner, combining the results of the interviews and the expert panel with the findings of the literature search. Based on these results, we drafted a second version of the OA module of the ABCC-tool which was used for content validity.

### 2.2 Content validation

#### 2.2.1 Participants

For the content validation at least ten HPs of a UMC and the DCGP OA committee and ten patients with OA of a UMC were invited to answer questions regarding relevance, comprehensiveness and comprehensibility, based on the COSMIN methodology [33]. Included HPs had to encounter patients with OA regularly. Inclusion criteria for patients were; diagnosis of OA of the hip, knee, foot, ankle, wrist, or hand, or a combination of these; being able to understand and read Dutch; and being at least 18 years old. Patients with OA localized exclusively in the neck, back, shoulder and elbow were excluded in this study, because the diagnosis of OA of the neck and back is difficult due to the lack of adequate diagnostic criteria [34], while OA of the shoulder is often accompanied by additional rotator cuff pathology [35] and OA of the elbow has a low prevalence [36].

#### 2.2.2 Procedure

Rheumatologists, orthopedic surgeons and specialized GPs completed an online questionnaire, based on the COMSIN methodology [33]. First, we introduced the ABCC-tool and presented the generic and lifestyle questions. Second, we presented the three OA-specific questions for which the HPs had to score the relevance per question with answer options ranging from one (not relevant) to four (highly relevant) [37]. Finally, we asked the HPs about the comprehensiveness and comprehensibility for patients with OA with a yes or no question. There was also the opportunity to motivate their answer or comment on the questions.

Patients were individually interviewed face-to-face, to evaluate relevance, comprehensiveness and comprehensibility, based on the COSMIN methodology [33]. They were asked if they thought the OA-specific questions were important for patients with OA (yes/no), if any topic of burden of disease was missing (yes/no), and if the questions were formulated clearly (yes/no). In case they answered ‘no’, they had to motivate their answer. At the end, there was an open question for feedback on the total questionnaire.

#### 2.2.3 Analysis

The item-content validity index (I-CVI) was used to assess content validity, using cut-off points, per question, for HPs. HPs scored each OA-specific question between one and four on relevance for OA patients (1 ​= ​not relevant, 2 ​= ​somewhat relevant, 3 ​= ​quite relevant, 4 ​= ​highly relevant). I-CVI scores were calculated by dividing the number of HPs scoring three or four by the total number of HPs in this study. Pre-set cut-off points between 0.00 and 1.00 were used to determine content validity. For example, when at least nine experts were interviewed, scores higher than 0.78, meaning that 78% of the experts regarded the questions valid, were seen as excellent. However, the more raters, the lower the cut-off point to reach content validity [[37], [38], [39]]. Patients rated the comprehensiveness and comprehensibility of the questions with a dichotomous answer option of yes or no.

### 2.3 Ethics

Informed consent was collected. No ethical approval was required according to the Dutch government. This study was approved by the Medical Ethics Committee Zuyderland Heerlen, the Netherlands (METCZ20210040).

## 3 Results

### 3.1 Development

The expert panel believed that developing the OA module for the ABCC-tool could improve OA care with a more personalized approach based on shared decision-making, by increasing self-management and self-reliance. In addition, HPs missed an instrument to monitor and quantify OA burden in current practice.

Regarding the interviews, we reached data saturation after interviewing thirteen patients with OA (twelve females) and nine HPs (three GPs, two physical therapists, one rehabilitation specialist, one rheumatologist and two orthopedic surgeons). The number of affected joints ranged from one to six, and nine patients (69%) had one or more comorbidities. The most affected joints were the knee (n ​= ​8) and foot (n ​= ​7).

HPs focused only on the physical burden of disease (pain and restriction in movement) and unhealthy lifestyle choices. However, patients mentioned physical (pain, joint stiffness and fatigue), emotional (kinesiophobia, concerns about the future and mood swings) and social (incomprehension from friends, family, and HPs) aspects as burden of disease. One patient said: “*You get a prescription for physically recovering. But mentally, nobody asks how you feel. … It is more than just a physical disease”*. Patients experienced the restrictions in daily life, work, hobbies, social life, etc. as the main consequence of OA, because of the limitations in movement. Most patients were not interested in conversations about lifestyle.

Based on the literature search, eight questionnaires were assessed to look for additional symptoms that may not have emerged from the interviews: Disability of the Arm, Shoulder and Hand Questionnaire (DASH), Foot and Ankle Outcome Score (FAOS), Functional Index of Hand OsteoArthritis (FIHOA), Hip Disability and Osteoarthritis Outcome Score (HOOS), Knee Injury and Osteoarthritis Outcome (KOOS), Michigan Hand Outcomes Questionnaire (MHQ), Score for the Assessment and Quantification of Chronic Rheumatoid Affection of the Hands (SACRAH), and the Western Ontario and McMaster Universities Osteoarthritis Index (WOMAC). Seven of them addressed additional questions about specific movements (e.g. walking stairs, cooking, and writing). Also, we identified questions about muscle weakness, tingling and QoL [[40], [41], [42], [43], [44], [45], [46], [47]]. Scoping the websites from ReumaNederland and the DCGP also revealed joint crepitus, and swelling and instability as OA related complaints [30,32].

Based on these three sources a first version of the OA module for the ABCC-tool was drafted, including two questions, i.e. pain and kinesiophobia. Questions and answer options were formulated the same way as the existing generic questions in the ABCC-tool (language level B1 and to be answered on a 7 point-Likert scale).

Twenty-seven patient with OA reviewed the first draft of the OA module. The question regarding kinesiophobia had some linguistic ambiguities. The question lacked clarity at the B1 language proficiency level and was therefore re-formulated. Additionally, the absence of a question concerning joint stiffness caught attention during the review process. Reviewing existing OA questionnaires in literature revealed consistent inclusion of a question about joint stiffness. Therefore, it was determined to incorporate an additional question addressing this aspect into the module. No comments were made about the answer options. Table 1 presents the three questions of the novel OA module of the ABCC-tool, which were used to assess content validity.

**Table 1:** Questions in the OA module of the ABCC-tool.

In the past month, to what extent …	0 Not at all	1 Very slightly	2 Slightly	3 Moderately	4 Very	5 Extremely	6 Totally
did you suffer from **pain**?	□	□	□	□	□	□	□
did you **avoid activities** to prevent an increase in pain?	□	□	□	□	□	□	□
did you suffer from **joint stiffness** (the feeling that the joint does not move smoothly)?	□	□	□	□	□	□	□

### 3.2 Content validation

**Fig. 2:**
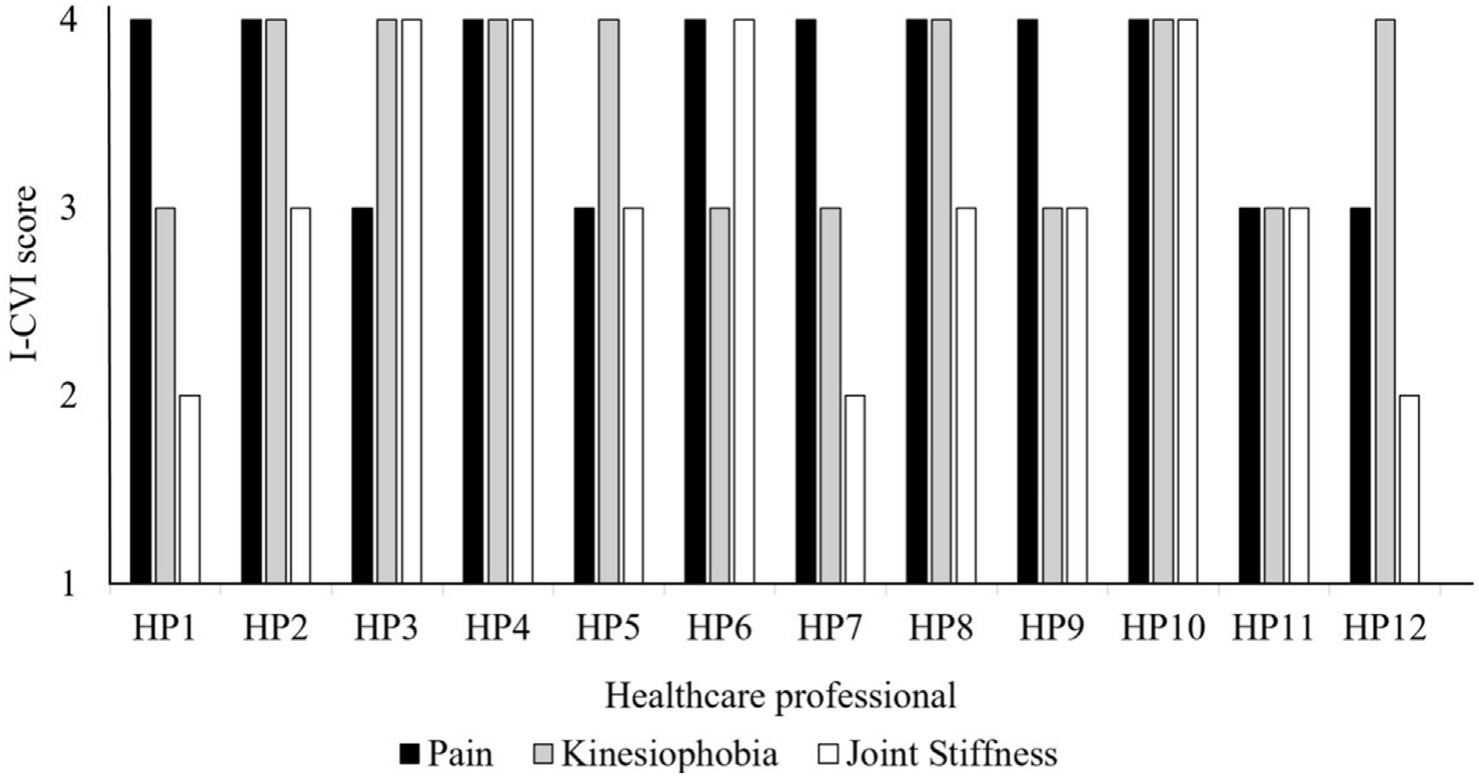
I-CVI scores per HP for pain, kinesiophobia and joint stiffness.

Twelve HPs (four specialized GPs, six rheumatologists and two orthopedic surgeons) completed the online questionnaire, while two did not complete the questionnaire and were considered lost to follow-up. The I-CVI score for both pain and kinesiophobia was 1.0, and 0.75 for joint stiffness. Individual scores per HP are presented in Fig. 2. HPs commented that pain and kinesiophobia were considered the major burden of disease for patients with OA, while joint stiffness was considered less important as this typically is only present for a short period after starting to move.

Seventeen patients were interviewed of whom four patients had two affected joints, and two patients had at least three affected joints. For the questions pain, joint stiffness, and kinesiophobia: 94% of the patients (16/17) indicated that these questions are important for OA patients. Patients provided no comments addressing these questions. The OA module appeared to be complete and all questions were clear, except for one patient who misinterpreted the question about kinesiophobia and needed help from a partner. All demographic data are presented in Table 2.

**Table 2:** Demographic data content validity.

*Patients*	n ​= ​17
Age	
Median (years)	67.0
IQR^a^ (years)	59.0–71.5
Female/Male	11/6
Educational level^b^	
Low	4
Medium	10
High	3
Dutch native speaker	16
OA^c^	
Hip	2
Knee	11
Foot/ankle	3
Hand/wrist	4
Other^d^ (shoulder, elbow, back, neck)	4
Time since OA diagnosis	
Median (years)	5.0
IQR^a^ (years)	0.4–10.0
*Healthcare professionals*	n ​= ​12
Health profession	
Rheumatologist	6
Specialized GP	4
Orthopedic surgeon	2
Joint specialization^e^	
Hip	11
Knee	12
Foot/ankle	5
Hand/wrist	9
Other (shoulder, elbow, back, neck)	8

a Interquartile Range.

b Educational level: low ​= ​primary school and VMBO; medium ​= ​HAVO/VWO and MBO; high ​= ​HBO and WO [46].

c OA: six patients had OA in at least two or more joints.

d Patients with only OA in other joints (shoulder, elbow, neck, back) are excluded in this study.

e Joint specialization: all HPs are specialized in at least two joints.

## 4 Discussion

In this study, we developed the OA module of the ABCC-tool and conducted a content validity assessment.

The development of this tool was characterized by a triangular iterative process including interviews with OA patients and HPs in the field of OA to identify if there is a demand for an OA tool. Patients indicated that there is a need to improve OA care as current care is mainly focused on a physical level, while the emotional and social burdens are rarely taken into account. Furthermore, HPs would like to discuss unhealthy lifestyle choices, but patients indicate that they are often not interested in this topic. Different expectations about existing OA care and patients’ expectations raise the need to personalize OA care, based on shared-decision making. HPs also highlighted the need for an instrument to monitor OA.

The strength of the previous developed ABCC-tool with modules for asthma, COPD, DM2 and chronic heart failure is that it includes care on a physical, emotional and social level, and facilitates shared-decision making. Studying the effectiveness of the ABCC-tool, it showed that patient experienced quality of care and patient activation improves [21,24,48]. Therefore, it was deemed desirable to create an OA module for the ABCC-tool because comparable effects might be expected.

Our results show that pain, kinesiophobia and joint stiffness form the main burden of OA, and should be combined in an OA module to be added to the generic module and lifestyle questions. Although other OA symptoms emerged during our study, e.g. complaints with specific movements such as climbing stairs or writing, or muscle weakness, we did not include these symptoms in the OA module, because these limitations are joint specific [30,32,[40], [41], [42], [43], [44], [45], [46], [47],^49^]. Our aim was to develop a module for OA which is not focused on a single specific joint. In addition, patients also mentioned several general burden of disease, such as having concerns about their future or restrictions in work. These burden have already been addressed in the generic part of the ABCC-tool [21]. For that reason, they are not included in the novel OA module. Therefore, we incorporated pain, kinesiophobia and joint stiffness into the OA module of the ABCC-tool.

To assess the content validity, seventeen patients participated in short interviews. The current version of the novel OA module appeared to be comprehensive. Although one patient needed help form a partner to understand the kinesiophobia question, we considered this version as the final version that could be used for the assessment of the content validation. All three OA questions were scored as important by sixteen out of seventeen patients (94%). This level of consensus suggests that patients find these burdens of disease equally important. Furthermore, twelve HPs shared their opinions on the OA questions. A perfect I-CVI score of 1.0 was found for pain and kinesiophobia, and 0.75 for joint stiffness. That joint stiffness is not seen as a major burden of disease by HPs might be explained by the fact that there is no therapy for this symptom, and that it disappears mostly after half an hour after starting to move. To attain content validity, it is recommended to achieve an I-CVI score of at least 0.78 when at least nine raters assessed the questions [39]. However, as the number of raters increases, the cut-off point of content validity decreases. Nevertheless, in this study, twelve HPs assessed the OA module of the ABCC-tool and no precise content validity cut-off point is known for twelve raters. It is worth mentioning that our I-CVI score of 0.75 for joint stiffness came remarkably close to reaching content validity when there are nine raters, with only 0.03 below the recommended cut-off point [[37], [38], [39]]. A thoughtful decision is needed to evaluate this item in the OA module of the ABCC-tool, because patients mentioned that joint stiffness is an important item. In addition, Van de Stadt et al. found that hand OA patients with joint stiffness generally have more pain and a lower QoL than OA patients without joint stiffness in their hands [50]. Maly et al. found a lower self-efficacy for physical tasks with greater knee stiffness, in patients with knee OA [51]. HPs may perceive joint stiffness differently than patients, but our primary goal with the OA module of the ABCC-tool is to support patients in managing OA. Therefore, we have decided to leave the question in the OA module for the time being. Future studies on the construct validity will hopefully give more insights in a definitive decision.

This study has several strengths. First, the development of an OA module builds on the already developed and evaluated ABCC-tool. Besides, we used several sources to construct the content of the OA module. Using an iterative approach gave us the opportunity to go back and forth in adjusting both the interview guide and the content of the novel OA module. Additionally, for the content validation we were able to interview more patients and HPs than intended, providing us with more information about the validity. Lastly, we had a mixed patient group characterized by a range in age, different affected joints, with and without comorbidities, and different duration of the OA disease.

There were also some limitations. First, in the development phase the majority of participating patients was female (12/13 female), which might have led to bias. Gender disparities in OA are well-documented. For example, women typically score worse on symptoms (e.g. more pain), therapeutic outcomes and functional limitations than men [52,53]. However, to the best of our knowledge, there are no burden aspects of OA present that exclusively affects one gender over the other. Therefore, using the combination of interviews and literature, we belief we were able to identify all burden aspects of OA to develop the OA module. For assessing the content validity, more male patients were included (6/17 male), to check for comprehensiveness of the OA module. As a result, we believe that the ABCC-tool holds promise for all genders in future applications, pending a positive evaluation of its construct validity. Second, instead of selecting items from an item bank, based on statistical analyses, we included items based on the experience of patients and HPs. Given the purpose of the ABCC-tool, this seemed the most appropriate way of item selection, as the tool is intended to discuss experienced burden by patients. Third, I-CVI scores were not computed for patients due to the inherent difficulty most patients experienced in responding to simple yes-or-no questions, such as “Do you believe the question about pain is important for an OA patient?” Instead, patients answered the questions in the OA module (“Yes, I have lots of pain”). Nevertheless, we could compare I-CVI scores three and four of HPs to the answer ‘yes’ from patients, and the I-CVI scores one and two to the answer ‘no’.

For future research, psychometric properties should be assessed, such as construct validity and reliability before implementing this novel OA module into the ABCC-tool. This should also give more disclosure if joint stiffness should be incorporated in the OA module of the ABCC-tool or not. Furthermore, the OA module was tailored and tested specifically for patients diagnosed with OA affecting the hip, knee, ankle/foot, and wrist/hand. Patients with OA localized exclusively in the shoulder, elbow, neck, and back were excluded from this research. HPs warned us that it is hard to diagnose OA in these joints, and that it is unclear if the burdens are related to OA or other musculoskeletal conditions. For example, back pain is mostly classified as non-specific lower back pain. Future research should investigate if the OA module is suitable for all joints.

### 4.1 Conclusion

The novel OA module of the ABCC-tool is developed and assessed for content validity and consists of three disease-specific questions related to pain, kinesiophobia and joint stiffness. With this novel module, the ABCC-tool is more elaborated and useable for a larger population. Pain and kinesiophobia are found highly valid in this study. Joint stiffness is seen as a less relevant item by HPs because there is no therapy that can decrease this. However, patients mentioned they suffered from joint stiffness as much as from pain and kinesiophobia. Future work should assess construct validity and reliability before the novel module can be used in practice.

## Author contribution

VD, AGS, RO, OvS and TB had a substantial contribution to the conception and the developing of the OA module for the ABCC-tool. Data analysis of the content validation was done by VD. AGS, RO, TB and OvS had a supervisory role during the evaluation and content validation process. The first manuscripts was written by VD, which was critically evaluated by all authors. The final version was read and approved by all authors as well. All authors agreed on the content of the work related to accuracy and integrity.

## Role of the funding source

This study was funded by ReumaNederland (NSP22-1-303). The funding party had no role in study design, data collection, analysis, interpretation or manuscript writing.

## Declaration of competing interest

There are no conflicts of interest.
